# Genomic prediction using a reference population of multiple pure breeds and admixed individuals

**DOI:** 10.1186/s12711-021-00637-y

**Published:** 2021-05-31

**Authors:** Emre Karaman, Guosheng Su, Iola Croue, Mogens S. Lund

**Affiliations:** 1 Center for Quantitative Genetics and Genomics, Aarhus University, 8830 Tjele, Denmark; 2 ALLICE, 78350 Jouy-en-Josas, France

## Abstract

**Background:**

In dairy cattle populations in which crossbreeding has been used, animals show some level of diversity in their origins. In rotational crossbreeding, for instance, crossbred dams are mated with purebred sires from different pure breeds, and the genetic composition of crossbred animals is an admixture of the breeds included in the rotation. How to use the data of such individuals in genomic evaluations is still an open question. In this study, we aimed at providing methodologies for the use of data from crossbred individuals with an admixed genetic background together with data from multiple pure breeds, for the purpose of genomic evaluations for both purebred and crossbred animals. A three-breed rotational crossbreeding system was mimicked using simulations based on animals genotyped with the 50 K single nucleotide polymorphism (SNP) chip.

**Results:**

For purebred populations, within-breed genomic predictions generally led to higher accuracies than those from multi-breed predictions using combined data of pure breeds. Adding admixed population’s (MIX) data to the combined pure breed data considering MIX as a different breed led to higher accuracies. When prediction models were able to account for breed origin of alleles, accuracies were generally higher than those from combining all available data, depending on the correlation of quantitative trait loci (QTL) effects between the breeds. Accuracies varied when using SNP effects from any of the pure breeds to predict the breeding values of MIX. Using those breed-specific SNP effects that were estimated separately in each pure breed, while accounting for breed origin of alleles for the selection candidates of MIX, generally improved the accuracies. Models that are able to accommodate MIX data with the breed origin of alleles approach generally led to higher accuracies than models without breed origin of alleles, depending on the correlation of QTL effects between the breeds.

**Conclusions:**

Combining all available data, pure breeds’ and admixed population’s data, in a multi-breed reference population is beneficial for the estimation of breeding values for pure breeds with a small reference population. For MIX, such an approach can lead to higher accuracies than considering breed origin of alleles for the selection candidates, and using breed-specific SNP effects estimated separately in each pure breed. Including MIX data in the reference population of multiple breeds by considering the breed origin of alleles, accuracies can be further improved. Our findings are relevant for breeding programs in which crossbreeding is systematically applied, and also for populations that involve different subpopulations and between which exchange of genetic material is routine practice.

**Supplementary Information:**

The online version contains supplementary material available at 10.1186/s12711-021-00637-y.

## Background

Genomic evaluation facilitates the accurate selection of genetically superior individuals as early as their DNA samples are obtained [1]. Genetic progress by selection depends on the accuracy of prediction. For genomic prediction, it depends on the proportion of genetic variance that is explained by genome-wide single nucleotide polymorphisms (SNPs), and the accuracy with which the effect of those SNPs is estimated [2, 3]. Both factors are conditional on the linkage disequilibrium (LD) between SNPs and quantitative trait loci (QTL) [1–3].

For an accurate genomic prediction, a large population of individuals with both phenotypes and genotypes is needed, which may not be possible for all traits and/or all breeds [4–6]. In such cases, remedies would be to use SNP effects from another breed (a strategy known as across-breed prediction) with a large reference population, or to add data from other breeds (multi-breed prediction) to improve the accuracy of the estimates of SNP effects. However, accuracies of across-breed predictions are generally around zero, and combining data from multiple breeds has not notably improved accuracies in empirical studies [7–10].

When multiple breeds are combined to form a reference population, predictions rely on the SNP-QTL LD across breeds. However, LD may be different [11, 12], or the phase of the SNP and QTL alleles may be reversed [13] among the breeds, due to selection and genetic drift [9]. The QTL, or SNPs in high LD with QTL can be integrated into marker panels for genomic prediction with a multi-breed reference population [14] or for performing across-breed predictions [15]. Although this may alleviate the issue that SNP-QTL LD can differ between different breeds, it includes an implicit assumption that QTL effects are the same across breeds. This may not be true if, for instance, interactions between QTL and genetic background exist [10, 11]. Hence, it may be more appropriate to assume that QTL, and therefore SNP effects are different but correlated, rather than they are the same across breeds.

Crossbreeding emerges as an efficient strategy for dairy cattle breeding to achieve better productivity and robustness at the animal as well as the system level. The improved performance is due to the use of specific combining abilities and heterosis [16]. In dairy cattle populations, in which crossbreeding has been used, animals show different levels of diversity in their origins [11, 17]. On the one hand, in rotational crossbreeding, for instance, where crossbred dams are mated to purebred sires from different pure breeds, the genetic composition of crossbred animals is an admixture of the breeds included in the rotation. At each rotation cycle, depending on the breed of the sires used, admixture proportions of crossbred individuals change considerably [18]. On the other hand, the gene pool of some “purebred” populations may also contain a fraction of the genome from other breeds, because bulls are used across the breeds to some extent [19]. A prerequisite for a well-structured crossbreeding system is to have an efficient breeding plan within the pure breeds, as well as crossbred population. Because, a sufficient number of purebred bulls is required for the system, and genetic gain in the pure breeds should be maintained to ensure that the overall economical benefit over time is not negatively affected [20]. Nonetheless, genomic evaluations in dairy cattle are mostly carried out separately for each breed, and neither are cross breed data used nor are breeders getting genomic evaluations for their crossbred animals. Therefore, in some breeding programs it is necessary that genomic prediction models can accommodate a reference population including admixed individuals, as well as multiple pure breeds, allowing simultaneous evaluation of all selection candidates.

An appealing approach to make use of data of admixed individuals in genomic prediction is to incorporate breed proportions in genomic prediction models. Makgahlela et al. [11] extended the random regression model to account for interactions between marker effects and breed proportions, where the breed proportions were inferred from pedigree data in Nordic Red Dairy cattle. They reported that prediction accuracy can be higher if breed proportions are considered. Thomasen et al. [21] performed genomic predictions in Danish Jersey dairy cattle, and concluded that a model that accounts for breed proportions, estimated either from pedigree or markers, does not improve the accuracy of genomic predictions compared to a model that ignores them. There are at least two limitations with both [11, 21] approaches. First, a single measure of breed proportion may not be appropriate, because two individuals with exactly the same breed proportions may have very different patterns of admixture along their genome depending on which chromosomal region is inherited from which pure breed [22]. Second, the correlations between the breeds were assumed to be homogenous across the whole genome [21], or those correlations were even set to zero due to difficulties in the estimation [11].

In this article, we propose a methodology that is suitable for genomic prediction using a reference population of multiple purebred and admixed individuals. Through simulations, we investigated the impact of the correlation of QTL effects between the breeds, and the heritability of the trait on the accuracy of genomic prediction using different approaches: (i) treating the combined data as a single homogeneous population, (ii) considering breed-specific SNP effects with or without accounting for correlations between the breeds, and (iii) considering priors that lead to the use of region-specific correlations among the breeds.

## Methods

### Data simulation

#### Genotypes

Genotype data at 51,477 loci were available for animals from each of the three dairy cattle breeds: Danish Holstein (HOL), Swedish Red (RED) and Danish Jersey (JER), from which a subset of 1050 (HOL and RED) or 220 (JER) individuals formed the base populations for this study. The SNPs that were fixed for the same allele in all three breeds were removed. For computational reasons, only the SNPs (12,664) on first five chromosomes were considered. A plot summarising the principle component analysis of genomic relationships among all animals was depicted to assess the genetic relationships between the pure breeds (see Additional file 1: Figure S1). In order to establish a data set including multiple pure breeds (i.e., HOL, RED and JER) and an admixed population (hereafter, MIX), a rotational crossbreeding system was mimicked using simulations, that considered three cycles of rotation (Table 1) for nine generations. Using the same sets of base population genotype data, 10 replicates were generated.

**Table 1:** Parents of each simulated generation

Generation/population	HOL^a^	RED	JER	MIX
1^b^	$\text {HOL}_0^\text {M} \times \text {HOL}_0^\text {F}$	$\text {RED}_0^\text {M} \times \text {RED}_0^\text {F}$	$\text {JER}_0^\text {M} \times \text {JER}_0^\text {F}$	$\text {JER}_0^\text {M} \times \text {HOL}_0^\text {F}$
$\vdots$	$\vdots$	$\vdots$	$\vdots$	$\vdots$
6	$\text {HOL}_5^\text {M} \times \text {HOL}_5^\text {F}$	$\text {RED}_5^\text {M} \times \text {RED}_5^\text {F}$	$\text {JER}_5^\text {M} \times \text {JER}_5^\text {F}$	$\text {HOL}_5^\text {M} \times \text {MIX}_5^\text {F}$
7	$\text {HOL}_6^\text {M} \times \text {HOL}_6^\text {F}$	$\text {RED}_6^\text {M} \times \text {RED}_6^\text {F}$	$\text {JER}_6^\text {M} \times \text {JER}_6^\text {F}$	$\text {JER}_6^\text {M} \times \text {MIX}_6^\text {F}$
8	$\text {HOL}_7^\text {M} \times \text {HOL}_7^\text {F}$	$\text {RED}_7^\text {M} \times \text {RED}_7^\text {F}$	$\text {JER}_7^\text {M} \times \text {JER}_7^\text {F}$	$\text {RED}_7^\text {M} \times \text {MIX}_7^\text {F}$
9	$\text {HOL}_8^\text {M} \times \text {HOL}_8^\text {F}$	$\text {RED}_8^\text {M} \times \text {RED}_8^\text {F}$	$\text {JER}_8^\text {M} \times \text {JER}_8^\text {F}$	$\text {HOL}_8^\text {M} \times \text {MIX}_8^\text {F}$

^a^HOL, RED, JER and MIX: Danish Holstein, Swedish Red, Danish Jersey and admixed population respectively

^b^Subscripts denote the generation, and superscripts denote the sex, i.e., males (M) and females (F)

Simulations started with 1050 (HOL and RED) or 220 (JER) individuals (generation 0–G0), of which 50 (HOL and RED) or 20 (JER) were assigned as males and the rest as females. The purebred populations were generated by mating sires and dams from the same breed (Table 1). Population sizes and the number of males and females were kept constant at each of the simulated generations for HOL, RED and JER. This was achieved by mating 20 dams with the same sire, each mating yielding one offspring, except for one mating which yielded two offsprings, for the simulations of HOL and RED. In the simulation of the JER population, each sire was mated with 10 dams, where each mating yielded one offspring, except for one mating which yielded two offsprings.

The MIX in G1 was generated by mating sires from JER and dams from HOL of G0. The MIX in G2 was generated by mating sires from RED and dams from MIX of G1. Finally, one rotation cycle was completed by generating MIX in G3 by mating sires from HOL and dams from MIX of G2. The following generations of MIX were generated by mating sires from a pure breed, where the pure breed depended on the rotation cycle, with the dams from the MIX (Table 1). Population size and the number of males ($n=50$) and females ($n=1000$) were also kept constant at each of the simulated generations for MIX. When MIX individuals were mated with HOL or RED, the mating structure was similar to that in the pure breeds, whereas when MIX (or HOL) individuals were mated with JER, each JER sire was mated with 50 dams, where 2 or 3 matings per sire were replicated to retain the population size of MIX at 1050. Selection was not considered, and mating was completely at random.

The number of recombinations on each chromosome was determined using a random variable drawn from a Poisson distribution, under the assumption that the length of a chromosome in Morgan (we assumed 1 cM ~ 1 Mb) is the lambda parameter [23]. Recombination positions were sampled from a uniform distribution, and interference was ignored [23]. Mutation was not considered in the simulations.

#### Phenotypes

The total number of QTL was set at 250, which were selected randomly among the SNPs that satisfied $0.01<\text {MAF} \le 0.30$, where MAF is the minor allele frequency computed as follows. First, allele frequency at each locus ($p_l$) was computed for each breed, and then averaged over the breeds ($\bar{p_l}$), to avoid population sizes affecting allele frequencies. Second, MAF of each locus was computed as $min(\bar{p_l},1-\bar{p_l})$. The selection of QTL with $0.01<\text {MAF} \le 0.30$ ensured that the QTL were segregating with a lower MAF compared to SNPs, for the combined population at G0. Table 2 shows some descriptive statistics for SNPs and QTL for each pure breed at G0. The QTL were excluded from the final data set of SNPs. It should be noted that although G0 was common to all 10 replicates, and therefore, the SNPs that met the criteria to be selected as QTL were the same, the QTL or SNP sets did not fully overlap among the replicates due to randomised selection of QTL. The effects (explained below) of QTL were also simulated separately for each replicate.

**Table 2:** Some descriptive statistics^a^ on SNPs and QTL for each pure breed in the base population (Generation 0–G0)

	HOL^b^	RED	JER
Number of fixed QTL for the reference (alternative) allele	9 (0)	5 (0)	58 (1)
Number of fixed SNPs for the reference (alternative) allele	564 (2)	385 (14)	2281 (286)
Number of breed-specific QTL	3	4	1
Number of breed-specific SNPs	261	356	50
Average MAF of segregating QTL	0.17	0.16	0.16
Average MAF of segregating SNPs	0.23	0.23	0.22

^a^Average over 10 replicates

^b^HOL, RED and JER: Danish Holstein, Swedish Red and Danish Jersey dairy cattle, respectively

Even if additive and dominance effects of QTL are the same in different breeds, the difference in QTL allele frequencies may cause substitution effects of QTL [16] to differ among breeds, as well as genetic (co)variances. In this study, the substitution effects of QTL were simulated directly from a multivariate normal distribution for varying levels of correlations among the QTL effects of different breeds, i.e., correlations of 1.00, 0.50 or 0.25.

Each individual had two alleles (maternal and paternal alleles) at each locus, inherited from its dam and sire. The breed origin of each allele for all loci was traced back to pure breeds at G0, and was known without error. The breeding value of each individual *i* ($u_i$) across G0–G9 was generated as follows:

$$\begin{aligned} \text{u}_{i} = \sum _{k=1}^{250} \left[ Q^M_{ijk}*\alpha ^M_{jk} + Q^P_{ijk}*\alpha ^P_{jk}\right], \end{aligned}$$

where $Q^M_{ijk}$ and $Q^P_{ijk}$ are the number of copies (0 or 1) of an arbitrarily chosen allele *A* at QTL locus *k*, inherited from its dam and sire breed *j* (*j* = *H,R,J* for HOL, RED and JER, respectively), respectively. The $\alpha ^M_{jk}$ and $\alpha ^P_{jk}$ are the simulated QTL effects for locus *k*, in breed *j*. The QTL effects were scaled such that the mean of the breed-specific genetic variances (computed as the variance of breeding values) is 100 at G0. A random residual $e_{i}$ drawn from a normal distribution, $e_{i}\mid \sigma _e^2 \sim N(0,\sigma _e^2)$, was added to each animal’s breeding value to generate its phenotypic value. The size of $\sigma _e^2$ was determined according to the simulated heritabilities (explained later) and the mean genetic variance (100) over the breeds. The same value of $\sigma _e^2$ was used in all generations for all individuals.

True (simulated) genetic correlations between the breeds were computed from the genetic variances, $\sigma ^2_{u,j} = \sum _{k=1}^{250} 2p_{jk}\left( 1-p_{jk}\right) \sigma ^2_{\upalpha _j}$, and covariances, $\sigma _{u,jj'} = \sum _{k=1}^{250} \sqrt{2p_{jk}\left( 1-p_{jk}\right) 2p_{j'k}\left( 1-p_{j'k}\right) }\sigma _{\upalpha _{jj'}}$ (*j* = *H,R,J* and $j\ne j^\prime$) [24] at *k* QTL. The genetic correlations between HOL–RED, HOL–JER and RED–JER were 0.88, 0.75 and 0.78, respectively, for a correlation between QTL of 1.00, over 10 replicates and at G0. These genetic correlations were 0.45, 0.38 and 0.38 for a correlation between QTL of 0.50, and 0.22, 0.19 and 0.19 for a correlation between QTL of 0.25, respectively. The differences between QTL effect correlations and genetic correlations were due to the difference in QTL allele frequencies between the breeds. The correlations between QTL allele frequencies of HOL–RED, HOL–JER and RED–JER were 0.33, 0.22 and 0.41, respectively. The correlations between SNP allele frequencies were 0.47, 0.32 and 0.46. The QTL effect correlations of 0.50 and 0.25 are consistent with the reported genomic correlations (genetic correlations estimated based on available SNP sets) between some cattle breeds for milk [14, 25] and fat [14], respectively. Two levels of heritability were considered for each scenario of correlations, i.e., 0.40 and 0.05, which are of the same magnitude as those reported for milk production and fertility traits, respectively (e.g., [6]). Due to fixing the size of the residual variance across the breeds, heritabilities fluctuated around these mean values across the breeds. Averaged over the replicates and correlation scenarios, realized heritabilities for the two traits (i.e., traits with heritabilities of 0.40 and 0.05) were 0.43 and 0.06 for HOL, 0.42 and 0.05 for RED, and 0.35 and 0.04 for JER.

### Reference and validation populations

Generations 6,7 and 8 (G6–G8) were used to form reference populations, while generation 9 (G9) was used to form validation populations. Hence, 660 JER individuals, and 3150 individuals from each of the HOL, RED and MIX were available for forming reference populations to estimate SNP effects.

The SNP effects were estimated using different reference populations: (i) a single pure breed (separate for each breed, i.e., HOL, RED or JER), (ii) combined data of multiple pure breeds (HOL + RED + JER), and (iii) combined data of multiple pure breeds and admixed (MIX) individuals. The MIX dataset was either used as a different “breed”, assuming homogeneous SNP effects across all breeds (HOL + RED + JER + MIX), or truly treated as an admixed population considering the breed origin of alleles (BOA) approach and uncorrelated (uncor) or correlated (cor) SNP effects between the breeds (HOL + RED + JER + MIX uncor/cor) [27, 40, 56, 57].

The prediction of breeding values for each pure breed was performed using: (1) the estimated SNP effects from their own breed (within-breed prediction), (2) the estimated SNP effects from each of the other breeds (across-breed prediction), (3) the estimated SNP effects from a combined reference population (multi-breed prediction) and (4) the estimated SNP effects from a combined reference population considering the BOA approach. The breeding values were predicted by multiplying SNP effects with allele dosages, with (4) or without (1–3) considering breed origin of alleles. These same strategies (1–4) were used to predict the breeding values of admixed individuals, with the addition of fixed breed contributions in multi-breed prediction [see model (1) in the next section]. For the admixed individuals, SNP effects estimated separately using pure breed reference populations (HOL/RED/JER) were also used to predict breeding values, considering the BOA approach only for the validation animals (hereafter, pure-BOA). That is, the breed origin of each SNP allele was traced back to its pure breed population only for the validation population, and the number of counted alleles was multiplied by the breed-specific estimate of SNP effects of the pure breeds.

We classified the methods using only a single breed’s data in model training to estimate SNP effects as “pure” (also includes pure-BOA as explained above), multiple breeds data without considering breed origin of alleles as “combined”, and multiple breed’s and MIX data considering breed origin of alleles as “BOA”.

### Statistical models

#### Pure and combined

A simple approach for genomic prediction using a combined reference population of multiple pure breeds and/or admixed individuals is to assume that the marker effects are the same across breeds [26]. For this simple approach, when the data consisted of multiple breeds treated as a single homogeneous population (Combined), we used the following model:

1 $$\begin{aligned} {\mathbf {y}} = {\mathbf {1}}\mu +{{\mathbf {X}}}{{\mathbf {b}}}+{\mathbf {M}}\varvec{\upbeta }+{\mathbf {e}}. \end{aligned}$$

In the above equation, ${\mathbf {y}}$ is the vector of phenotypes $(n\times 1)$, ${\mathbf {1}}$ is a vector of 1s $(n\times 1)$, $\mu$ is the general mean, ${\mathbf {X}}$ is the matrix of breed proportions $(n\times 3)$ computed from SNP data, ${\mathbf {b}}$ is the vector of fixed breed effects $(3\times 1)$, ${\mathbf {M}}$ is the matrix of centered genotypes $(n\times l)$ where centering was based on the current allele frequencies in the combined data, $\varvec{\upbeta }$ is the vector of SNP effects, and ${\mathbf {e}}$ is the vector of residuals $(n\times 1)$. The value of *n* depends on the reference population size, and *l* is the number of SNPs. Model (1) was used without the breed proportions component ${{\mathbf {X}}}{{\mathbf {b}}}$ when the SNP effects were estimated separately for each pure breed (pure and pure-BOA).

#### BOA

Admixed breed’s data was used by extending the existing linear model proposed for simple 2-way crosses (e.g., [27]) to accommodate more than two pure breeds:

2 $$\begin{aligned} {\mathbf {y}}={\mathbf {1}}\mu +{\mathbf {M}}_{H}\varvec{\upbeta }_{H}+{\mathbf {M}}_{R}\varvec{\upbeta }_{R}+{\mathbf {M}}_{J} \varvec{\upbeta }_{J}+{\mathbf {e}}, \end{aligned}$$

where ${\mathbf {y}}$ is the vector of phenotypes $(n\times 1)$ of all animals, that is, both purebred and admixed animals, ${\mathbf {1}}$ is a vector of 1s $(n\times 1)$, $\mu$ is the general mean, ${\mathbf {M}}_H$, ${\mathbf {M}}_R$ and ${\mathbf {M}}_J$ are the matrices of breed specific allele content of SNPs $(n\times l)$ for HOL, RED and JER, respectively. The entry at a locus in, for instance ${\mathbf {M}}_H$, for an animal were the number (0, 1 or 2) of counted alleles *A* originated from HOL. That is, when the animal had no allele originating from HOL, or when a HOL animal had an *aa* genotype, the corresponding entry was zero. The same applied to matrices ${\mathbf {M}}_R$ and ${\mathbf {M}}_J$. The matrices were column centered prior to analysis. The $\varvec{\upbeta }_H$, $\varvec{\upbeta }_R$ and $\varvec{\upbeta }_J$ are vectors of SNP effects for HOL, RED and JER, respectively, and ${\mathbf {e}}$ is the vector of residuals.

### Bayesian analysis

A Bayesian approach was considered in the parameter estimation, which requires assigning prior distributions to the unknowns of the model. Analyses were carried out separately for each trait. To investigate the impact of assuming a heterogeneous (co)variance of SNP effects between different genome regions, three region sizes were considered based on a fixed number of SNPs; 1 SNP, 100 SNPs and the whole genome (WG). Region sizes of 1 SNP and WG can be regarded as BayesA and SNP-best linear unbiased prediction (SNP–BLUP) [1], (or equivalently genomic BLUP, GBLUP [28]) when using model (1), and extensions of them for multiple components (breeds) when using model (2), respectively. In BayesA, it is assumed that each SNP (1 SNP) follows a normal distribution with null mean and a locus-specific variance, while in GBLUP it is assumed that all SNPs (WG) have null means and a common variance. To consider the heterogeneous variance of SNP effects among different genome regions using model (1), the matrix of genotypes and vector of SNP effects were partitioned into *S* subsets each with $l_s$ loci ($s=1,\dots ,S$), and priors were assigned to each sub-vector of $\varvec{\upbeta }$: $\varvec{\upbeta }_{s}\mid \sigma _{s}^{2}\sim N({\mathbf {0}},{\mathbf {I}}\sigma _{s}^{2})$ [29, 30]. The $\sigma _{s}^{2}$(s) were further assigned a scaled inverse chi-square prior with a number of degrees of freedom (*df*) and a scale parameter (*S*): $\sigma _{s}^{2}\mid df,S \sim \chi^{-2}(df,S)$. The values of hyper-parameters will be explained later.

In the analyses using model (2), all genotype matrices and vectors of SNP effects were also partitioned into *S* subsets each with $l_s$ loci. A normal distribution prior was assigned for each sub-vector of SNP effects for population *j* (*j* = *H,R,J*): $\varvec{\upbeta }_{j,s}\mid \sigma _{j,s}^{2}\sim N({\mathbf {0}},{\mathbf {I}}\sigma _{j,s}^{2})$. Hence, the SNP effects were breed-specific and uncorrelated across the breeds. That is, the genetic correlations between the breeds were assumed to be zero. The $\sigma _{j,s}^{2}$(s) were further assigned a scaled inverse chi-square prior with a number of degrees of freedom ($df_j$) and a scale $(S_j)$ parameter: $\sigma _{j,s}^{2}\mid df_{j},S_{j} \sim \chi^{-2}(df_j,S_j)$. Using model (2), priors were also assigned such that the marker effects were breed-specific, but correlated between the breeds. That is, a multivariate normal distribution was assigned for each sub-vector of SNP effects: $$ \begin{bmatrix} \varvec{\upbeta }_{H,s}\, \varvec{\upbeta }_{R,s}\, \varvec{\upbeta }_{J,s} \end{bmatrix}^\prime \mid {\mathbf {B}}_s \sim N \Big ( {\mathbf {0}}, {\mathbf {B}}_s \otimes {\mathbf {I}} \Big ) $$, where ${\mathbf {I}}$ is an identity matrix of size equal to $l_s$ if $l_{s}>1$ or a scalar of 1 if $l_{s}=1$.

$$\begin{aligned} {\mathbf {B}}_s = \begin{bmatrix} \sigma ^2_{H,s}&{}\sigma _{HR,s}&{}\sigma _{HJ,s} \\ \sigma _{RH,s}&{}\sigma ^2_{R,s}&{}\sigma _{RJ,s} \\ \sigma _{JH,s}&{}\sigma _{JR,s}&{}\sigma ^2_{J,s} \end{bmatrix} \end{aligned}$$

The diagonals of ${\mathbf {B}}_{s}$ are the breed-specific SNP variances, and the off-diagonals are SNP covariances between the breeds. The ${\mathbf {B}}_{s}$ was assumed to follow an inverted Wishart distribution with a shape $(v_{B})$ and a scale $({\mathbf {V}}_{B})$ parameter: ${\mathbf {B}}_{s}\mid v_{B},{\mathbf {V}}_{B}\sim IW(v_{B},{\mathbf {V}}_{B})$.

In both models (1) and (2), residuals were assigned a univariate normal prior, $e_{i}\mid \sigma _{e}^{2}\sim N(0,\sigma _{e}^{2})$, and the variance $\sigma _{e}^{2}$ was assigned a scaled inverse chi-square prior with a number of degrees of freedom $(df_e)$ and a scale $(S_e)$ parameter: $\sigma _{e}^{2}\mid df_{e},S_{e} \sim \chi^{-2}(df_e,S_e)$. Fixed effects were assigned flat priors.

The hyper-parameters of the prior distributions for the variance components were derived from the simulated genetic (co)variances and residual variances at G0 as follows. For the analysis using model (2) assuming independent SNP effects among the breeds, $df_j=4$ and $\,S_{j}=\frac{\sigma _{j,s}^{2}\left( df_{j}-2\right) }{df_{j}},\,\text {where}\;\sigma_{j,s}^{2}=\frac{\sigma _{u,j}^{2}}{\sum 2p_{j,l}\left( 1-p_{j,l}\right) }$ [31]. Here, $\sigma _{u,j}^{2}$ is the genetic variance for breed *j*, and $p_{j,l}$ is the allele frequency of *l*th SNP in breed *j*. Only one $\,S_{j}$ was required for the analysis using model (1), which was computed using $\sigma _{u,j}^{2}$ (pure breed analysis) or the mean value of $\sigma _{u,j}^{2}$ over the breeds (combined analysis), and $df=4$. For the analysis using model (2) assuming correlated SNP effects between the breeds, ${\mathbf {V}}_{B}=(v_{B}-3-1){\mathbf {B}}$ where $v_{B}=6$, and

$$\begin{aligned} {\mathbf {B}}= \begin{bmatrix} \frac{\sigma ^2_{u,H}}{\sum 2p_{H,l}\left( 1-p_{H,l}\right) }&{}\frac{\sigma _{{u,HR}}}{\sum \sqrt{2p_{H,l}\left( 1-p_{H,l}\right) }\sqrt{2p_{R,l}\left( 1-p_{R,l}\right) }}&{}\frac{\sigma _{{u,HJ}}}{\sum \sqrt{2p_{H,l}\left( 1-p_{H,l}\right) }\sqrt{2p_{J,l}\left( 1-p_{J,l}\right) }} \\ \frac{\sigma _{{u,RH}}}{\sum \sqrt{2p_{R,l}\left( 1-p_{R,l}\right) }\sqrt{2p_{H,l}\left( 1-p_{H,l}\right) }}&{}\frac{\sigma ^2_{{u,R}}}{\sum 2p_{R,l}\left( 1-p_{R,l}\right) }&{}\frac{\sigma _{{u,RJ}}}{\sum \sqrt{2p_{R,l}\left( 1-p_{R,l}\right) }\sqrt{2p_{J,l}\left( 1-p_{J,l}\right) }} \\ \frac{\sigma _{u,JH}}{\sum \sqrt{2p_{J,l}\left( 1-p_{J,l}\right) }\sqrt{2p_{H,l}\left( 1-p_{H,l}\right) }}&{}\frac{\sigma _{{u,JR}}}{\sum \sqrt{2p_{J,l}\left( 1-p_{J,l}\right) }\sqrt{2p_{R,l}\left( 1-p_{R,l}\right) }}&{}\frac{\sigma ^2_{u,J}}{\sum 2p_{J,l}\left( 1-p_{J,l}\right) } \end{bmatrix}. \end{aligned}$$

In the above equation, $\sigma ^2_{u,j}$ and $\sigma _{{u,jj'}}$ (*j* = *H,R,J* and $j\ne j^\prime$) are genetic variances and covariances, respectively. For residual variances, $df_e=4$ and $S_{e}=\frac{\sigma _{e}^{2}\left( df_{e}-2\right) }{df_{e}}$, where $\sigma _{e}^{2}$ is the residual variance at G0.

The Markov-chain Monte Carlo (McMC) algorithm was used to obtain samples of each parameter from its full-conditional posterior distribution. The chain length for the analyses consisted of 50,000 cycles, of which the first 10,000 were discarded as burn-in. Every 10th sample of the post burn-in cycles was kept for posterior analysis, yielding 4000 posterior samples. The mean value of the posterior samples was used as the estimate of each parameter. All the analyses were performed using self-written scripts in Julia [32].

### Prediction accuracy

Prediction accuracy was assessed as the correlation between true and predicted breeding values of validation individuals (1050 individuals for HOL, RED, MIX, and 220 individuals for JER) at G9. The accuracies of prediction using different data sets and models to estimate SNP effects were compared for each trait, QTL correlation and region size, separately. The accuracies of prediction for different region sizes were compared for each data set and model, trait and QTL correlation, separately. All comparisons were performed using a two-sided paired t-test, for which accuracies were paired across each replicate for the same validation population. A Bonferroni correction was used to control the type 1 error rate of 0.05.

## Results

**Fig. 1:**
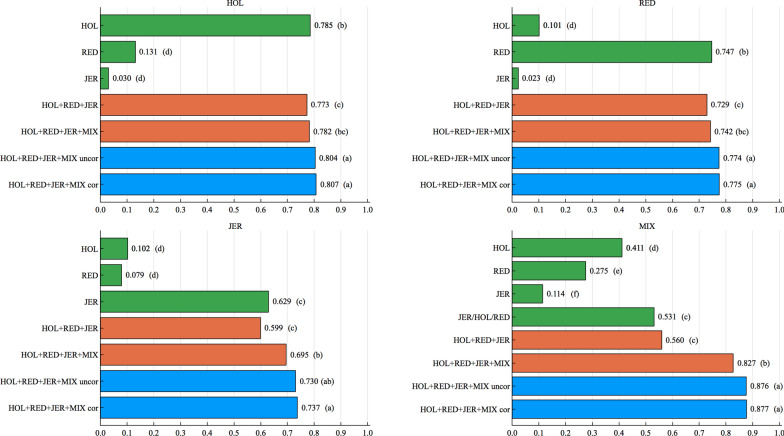
Accuracies (horizontal axis) for the high heritability trait ($h^2=0.40$) in the scenario with a QTL correlation of 0.50, using different data sets or models (vertical axis), for a region size of 1 SNP. The predicted population is given on top of each plot. Letters in parentheses stand for the significance tests

**Fig. 2:**
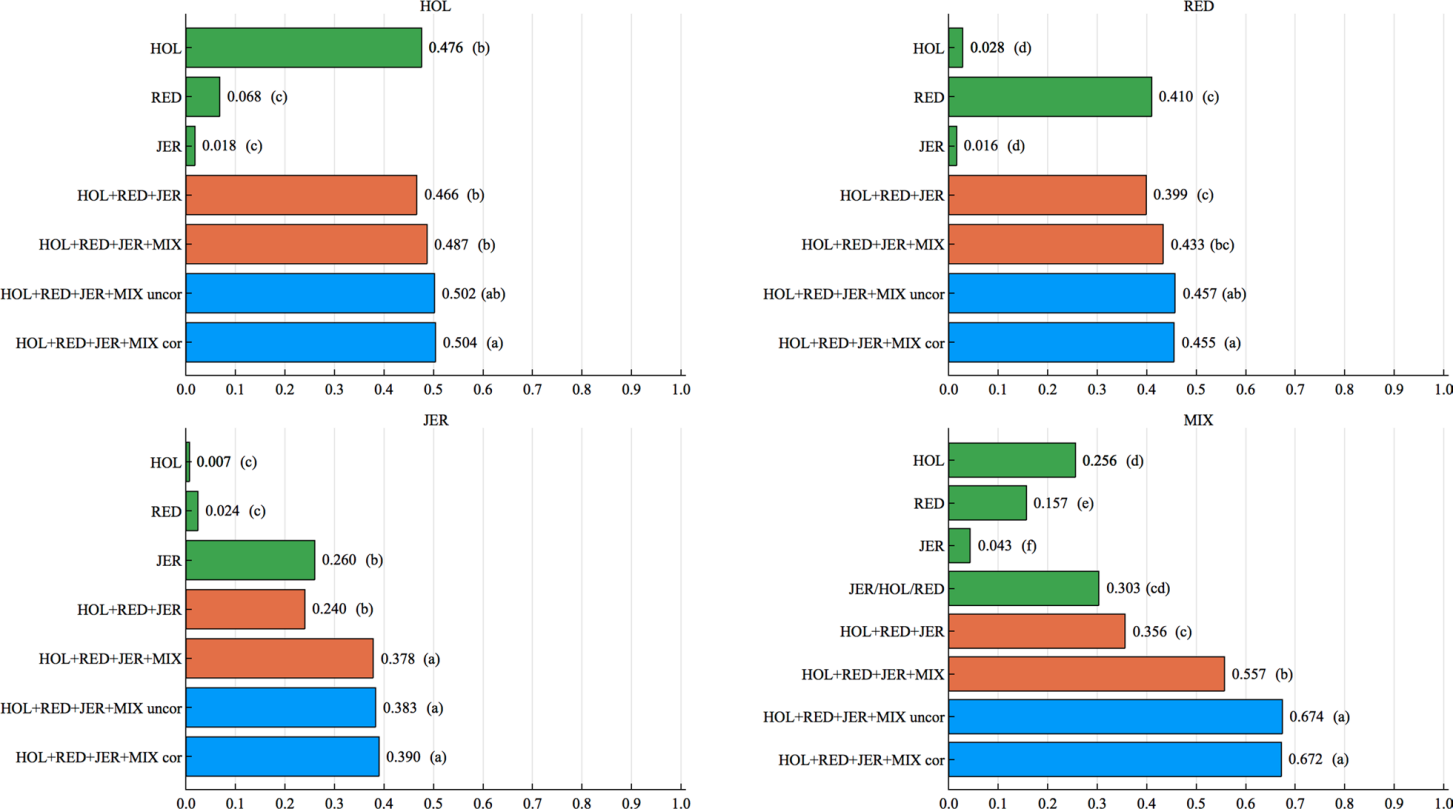
Accuracies (horizontal axis) for the low heritability trait ($h^2=0.05$) in the scenario with a QTL correlation of 0.50, using different data sets or models (vertical axis) for a region size of 1 SNP. The predicted population is given on top of each plot. Letters in parentheses stand for the significance tests

Accuracies for all scenarios and all region sizes are given in Additional file 2: Tables S1–S4. For readability, only the core results obtained with a QTL effect correlation of 0.50 are presented in the main text. Accuracies were higher for a high heritability trait than for a low heritability trait (Figs. 1 and 2). Within-breed predictions for breeds with large reference populations (HOL and RED) were more accurate than for a breed with a small reference population (JER). For the high heritability trait, within-breed predictions for HOL, RED and JER were 0.785, 0.747 and 0.629, respectively, when the region size was 1 SNP (Fig. 1). For this high heritability trait, combining data from multiple pure breeds (HOL + RED + JER) assuming homogenous SNP effects (multi-breed prediction) did not improve, or even decreased (but not always significantly) the accuracies for all breeds. Including the admixed population’s (MIX) data in multi-breed prediction, considering MIX as a different breed (HOL + RED + JER + MIX), yielded higher accuracies compared with combining only the data from pure breeds, and similar to or higher accuracies than using the single breed data alone (within-breed prediction), for genomic prediction of JER. When prediction models were able to accommodate data of admixed individuals by accounting for breed origin of alleles (HOL + RED + JER + MIX uncor/cor), accuracies were generally improved compared to combining all available data, but this depended on the correlation scenario. Across-breed predictions yielded much lower accuracies than within-breed predictions.

**Fig. 3:**
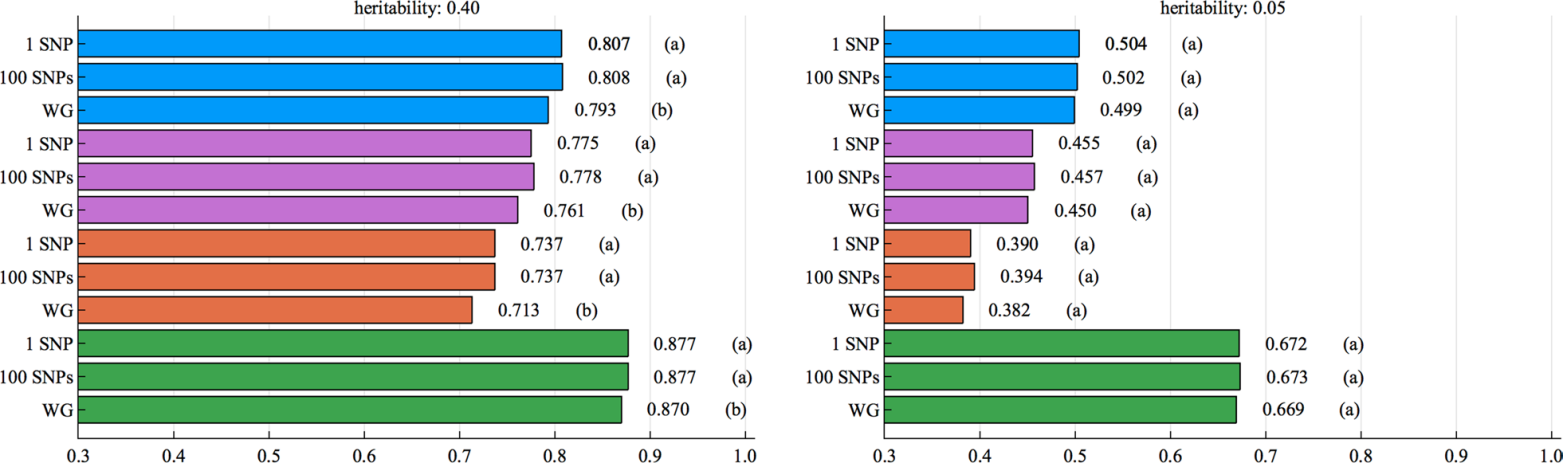
Accuracies (horizontal axis) for the high ($h^2=0.40$, left figure) and the low ($h^2=0.05$, right figure) heritability trait using the BOA model with correlated SNP effects and different region sizes (vertical axis), in the scenario with a QTL correlation of 0.50. Blue, purple, red and green bars represent accuracies from HOL, RED, JER and MIX, respectively. Letters in parentheses stand for the significance tests

Accuracies were lowest when using SNP effects from any of the pure breeds to predict the breeding values of admixed individuals. For the high heritability trait, predictions using SNP effects of HOL, RED and JER yielded accuracies of 0.411, 0.275 and 0.114, respectively, when the region size was 1 SNP (Fig. 1). For the same scenario and region size, estimating SNP effects separately for each breed, but accounting for breed origin of alleles in the prediction of breeding values (HOL/RED/JER) of MIX, improved accuracy up to 0.531. Combining MIX data with pure breeds’ data assuming common SNP effects for all breeds (HOL+RED+JER+MIX), improved accuracies compared to combining only three pure breeds’ data (HOL + RED + JER) for the accuracy of admixed individuals (0.827 vs 0.560). Models that can use MIX data with breed origin of alleles (HOL + RED + JER + MIX uncor/cor), improved accuracies compared to combining all available data, i.e., combining all purebred data or all purebred and admixed individuals’ data, although it depended on the correlation in the QTL scenario (see Additional file 2: Tables S1–S4). Accounting (0.877) or not (0.876) for correlations between the SNP effects of different pure breeds did not make any difference (Fig. 1, MIX). For a QTL correlation of 1.00 and predictions in MIX, (HOL+RED+JER+MIX) led to higher accuracies than (HOL + RED + JER + MIX uncor). Among the different region sizes considered here (1 SNP, 100 SNPs and the whole genome (WG)), the WG region size generally yielded the lowest accuracies for pure breeds and the admixed population (Fig. 3).

The efficiency of the methods considering breed origin of alleles in model training became more apparent as the correlation of the simulated QTL effects between the breeds decreased (see Additional file 2: Tables S1–S4). For the high heritability trait and purebred populations, accuracies for (HOL + RED + JER + MIX uncor/cor) were significantly higher than those for (HOL + RED + JER + MIX) in the scenario with a QTL effect correlation of 0.25. For the MIX population, (HOL + RED + JER + MIX uncor/cor) yielded significantly higher accuracies than (HOL + RED + JER + MIX), for QTL effect correlation of 0.25, and for both traits.

## Discussion

### Within- and across-breed predictions

A simple approach for avoiding the unfavourable impact of the difference in marker effects among different purebred populations is to carry out separate evaluations for each of those pure breeds, as is the case for genomic evaluations in many countries [19]. Such an approach, however, comes with the cost of a potential loss of data information, and therefore, in the accuracy of SNP effect estimation. This is a limitation for genetic improvement in populations with a small genomic reference population. In this study, accuracies from within-breed predictions were higher for HOL and RED, compared to JER. Although there could be other reasons, one explanation is the small reference population size (660 vs 3150) set for JER. The accuracies for pure breeds differed between the two heritability levels for any QTL effect correlation scenario, with accuracies being higher for the high heritability trait than for the low heritability trait. The fact that genomic prediction accuracy is higher with large reference populations and/or for a high heritability trait has been reported in many other studies [3, 5, 33–35]. It should be noted that the accuracies for the same heritability level fluctuated slightly for different QTL effect correlation scenarios, because QTL effects were simulated using different multivariate normal distributions (the covariance matrices differed) for those scenarios.

Using SNP effects of one pure breed to predict the breeding values of individuals of the other breeds (across-breed prediction), yielded much lower accuracies than within-breed predictions. This was true even when the simulated QTL effects had a correlation of 1.00. It should be noted that, although the simulated QTL effects were identical in the scenario with a correlation of 1.00, some QTL were not segregating in each breed (Table 2). The results for across-breed prediction are in line with the study of Steyn et al. [36] in which several breeds were simulated assuming identical QTL effects, but across-breed predictions were poor. Studies using real data also showed that using data from one breed to predict breeding values in the other breeds results in accuracies as low as zero (e.g., [9, 10]). The prediction accuracy of MIX generally reflected the expected breed proportions of the validation individuals. Using SNP effects from HOL, for instance, led to the highest prediction accuracies for MIX, as HOL was the most recent ancestor population for MIX, and therefore, MIX individuals had a higher proportion of their genome from HOL.

For within-breed predictions, both family relationships and LD between SNPs and QTL contribute to accuracy [37–39]. For across-breed prediction, the relationships of the individuals of the target breed with the individuals in the reference population are lower than those with the members of the target breed. The relative contributions of the two factors, family relationships and LD, to accuracy of breeding value estimation were not studied as it was not within the scope of this paper. If we rely on the argument that low across-breed prediction accuracy is due to differences in LD patterns among the breeds, i.e., the differences in LD or the phase of the SNP and QTL alleles, then across-breed prediction can not compete with within-breed prediction [15], even for closely-related breeds. In addition to LD patterns, it is also possible that QTL effects and/or QTL allele frequencies differ among the breeds, while some QTL may only segregate in one breed [25, 40]. Needless to say, even if the QTL properties were the same among the breeds, SNP effects would still be different to the extent that LD between SNPs and QTL differs between them [11, 12, 30, 41].

Although the simulated traits in this study were relatively polygenic, the variance structure at the SNP level may be different from that at the QTL level along the genome [42, 43], favouring models that can accommodate such heterogeneity [30, 44, 45]. The SNP panels tend to include SNPs with a high minor allele frequency (MAF), while generally the QTL have a low MAF [46, 47]. The LD between the two sets, SNPs and QTL, can not be perfect if their MAF differs. Because the SNPs within a region of the genome are likely inherited together, and also likely to be in LD with the same QTL, they may collectively capture the genetic variance at the QTL [29, 45, 48]. Hence, assuming a common variance for groups of adjacent SNPs is reasonable, while it allows more Bayesian learning compared to assuming variance specific to every single SNP [49]. For regions with a size larger than an optimum level, the advantage of grouping adjacent SNPs will start to disappear as the assumption on (co)variance will approach that of the whole genome region size (WG).

For a high heritability trait and purebred analysis, accuracies obtained for different region sizes were generally ranked as 100 SNPs > 1 SNP > WG. It was shown earlier by simulations [30, 35] and real data analysis that assigning priors to groups of SNPs may improve accuracies [44, 45] compared to assigning a common prior for all SNPs. However, in a recent study, Liu et al., [50] reported negligible differences between several region sizes (one, 30, or 100 SNPs, and WG) for milk production and fertility traits in Danish Jersey, and using a model which is nearly identical to our model (1).

### Combined data from multiple pure breeds

If the studied population is small, it might be challenging to establish a large reference population, and in turn the accuracy of genomic prediction might also be limited [6]. For breeds with a limited reference population size, incorporating data from other breeds may yield higher accuracies [26, 40, 51], although it depends on the relatedness between those breeds [9, 19]. When HOL and RED individuals were included in the reference population of JER (HOL+RED+JER), accuracies generally dropped. Similarly, using the combined reference population, accuracies for HOL and RED also generally dropped, but less compared to those for JER. When multiple purebred populations are combined to form a reference population, SNP effects are dominated by the breeds that contribute more to the reference population. This may cause prediction models to pick up only the effect of SNPs that are in LD with QTL in all breeds, and/or only in the largest population, but not the effect of SNPs specific to small populations [14]. We had additional simulations where all breeds had the same number of individuals in the reference population (3150 for each), which resulted in accuracies for JER being also high and getting less affected from the joint analysis, as HOL and RED (results not given). These imply that the proportion of each single breed in a combined reference population of multiple breeds is important to achieve a sufficient accuracy for each breed, particularly when the breeds are genetically distant. This was more formally investigated in [40] using a high-density SNP chip ($\sim$ 600,000 SNPs), where one of the two breeds (Holstein and Jersey) that formed a joint reference population had varying sizes, 0, 100, 500 or 2000 animals, while the size of the other breed was kept constant at 2000 animals. As the number of individuals of a breed in the joint reference population decreased, accuracies for the candidates of the same breed also decreased [40].

In a study based on real genotypes of imputed sequence variants ($\sim 1$ million SNPs), van den Berg et al. [52] simulated phenotypes for four dairy cattle breeds using identical QTL effects. They reported generally higher accuracies for multi-breed predictions, compared to within-breed predictions. In our scenario with a QTL effect correlation of 1.00, the difference in the accuracies from within- and multi-breed predictions were smaller compared to other (lower) QTL effect correlation scenarios. At long distances in the genome, LD differs between species and also between different cattle breeds, whereas it is relatively consistent at short distances [3]. The standard SNP sets, such as the one used here, are not sufficient to include all the SNPs that are in high LD with QTL across the breeds. Moreover, we selected QTL such that they had a relatively low MAF compared to SNPs, and this has an impact on LD between QTL and SNPs, whereas (randomly selected) QTL were included in the SNP set in [52]. These may partially explain why multi-breed genomic predictions generally had lower accuracies than within-breed predictions even when the simulated QTL effects were identical, compared to the findings of [52].

For the analysis of data consisting of multiple breeds (or lines or populations), an appealing strategy is to apply multi-trait methods where the same trait in different breeds is considered as different but two correlated traits, e.g. [8, 25]. In those applications of multi-breed genomic prediction, however, a homogeneous genomic correlation was assumed across the genome, for pairs of breeds. Lehermeier et al. [41] applied a multivariate modelling approach, which is flexible in that both marker effects and their (co)variances are allowed to differ among multiple breeds, but it still assumes a homogenous correlation across the genome of breed pairs. Chen et al. [53] proposed a method which allows the estimation of SNP effects that are specific to each breed while accounting for heterogenous (co)variances across the genome. Their method, however, applies a variable selection procedure that aims at pinpointing the SNPs that have an effect in all the breeds involved, leaving out the SNPs that have an effect on only one or a subset of the breeds. It was further extended by Calus et al. [10] so as to accommodate also the selection of SNPs that are breed-specific. Nevertheless, both methods [10, 53], make limited use of the correlated information in the data, because, regardless of how the SNPs that are to be included in the model are selected, their effects are estimated separately within each breed. Furthermore, all those multi-trait approaches are pertained to situations where individuals can be assigned to certain pure breeds, and are not able to accommodate data of individuals with an admixed genetic background.

### Genomic prediction including data from admixed individuals

If a large amount of commercial farm data for admixed populations becomes available, it can help to improve selection accuracy by expanding the data size for each pure breed population [12]. Such data can also allow to exploit heterosis due to dominance and can accelerate performance of crossbred animals in commercial farms [54]. How to use those data in genomic evaluations is still an open question. Naturally, all purebred and admixed individual data can be combined together, when homogeneous SNP effects are assumed.

Including the data of an admixed population (MIX) along with the data of pure breeds in the reference population led to higher accuracies than the combined reference population of pure breeds. The JER benefited relatively more from adding MIX data. Because we mimicked a rotational cross breeding system, at each generation, admixed population dams were sired by a purebred individual. Consequently, when an admixed female was mated with a purebred male, the offspring had an entire paternal chromosome from a pure breed, and a maternal chromosome including large chunks of the (i) admixture of all breeds and (ii) the pure breed of the maternal grand-sire. This means that, at each generation following G1, pure breeds were not equally represented in the genome of admixed individuals. Consider a single admixed individual at generation 6. That individual has an expected breed composition for a maternal chromosome of roughly 28% JER, 16% HOL, and 56% RED, and for a paternal chromosome of %100 HOL. Those proportions change to 14% JER, 58% HOL, and 28% RED for a maternal chromosome, and 100% JER for a paternal chromosome at generation 7, and to 57% JER, 29% HOL, and 14% RED for a maternal chromosome, and 100% RED for a paternal chromosome at generation 8. Because a full rotation cycle of three generations (G6–G8) was considered when forming the reference populations, each pure breed was represented in the MIX data almost equally. Thereby, the reference population size increased almost equally for all breeds by adding MIX data to the combined data of three breeds, HOL + RED + JER + MIX. As one would expect, JER benefited more from this increase in data size, since it is the breed with the smallest pure breed reference population. It should be noted that the validation individuals of RED had the grand-sires which were also the sires of MIX at G8, and G8 was included in the reference population. Hence, although the data size increased almost equally for each breed, the added value of this may not be equal for all the breeds.

More elaborative ways to include individuals with admixed genetic background in the genomic evaluations, were proposed. Makgahlela et al. [11] fitted a multi-trait random regression model to account for interactions between marker effects and breed proportions, where the breed proportions were inferred from pedigree data in Nordic Red Dairy cattle. They reported, for some traits, higher prediction accuracies for the model accounting for breed proportions, than a GBLUP model treating the data as a single homogeneous population. Another example of admixture is admixture due to different populations, instead of breeds. Danish Jersey dairy cattle, for instance, include animals with different proportions of their genome from original Danish and US Jersey populations [19, 21]. Although both originate from a single breed, they have been separated long ago, and the persistency of phase was shown to differ between the two, particularly at long distances between loci [21]. Hence, the accuracy of genomic prediction for Danish Jersey may not be challenged only by the small reference population size, but also by its admixed population structure. In order to overcome the negative impact of admixed population structure in Danish Jersey on genomic prediction accuracy, Thomasen et al. [21] applied a set of random regression models that included proportions of population origin for each animal. Contrariwise, Thomasen et al. [21] did not find any strong evidence that a model which accounts for proportions of population origin, estimated either from pedigree or markers, is superior to a model which ignores them. A possible explanation could be that admixture due to different breeds may be a more serious problem than admixture due to subpopulations of the same breed, in genomic prediction. Nevertheless, there are at least two limitations with both [11, 21] approaches. First, breed proportions of an individual were average values along their whole genome, which were computed based solely on pedigree or markers. This may not be appropriate, as two individuals with exactly the same breed proportions may have very different admixture patterns over their genome depending on which chromosomal region is inherited from which pure breed [21, 22]. Second, their models are somewhat restricted in that the correlations between the breeds were assumed to be homogenous across the whole genome [21], or those correlations were even set to zero to account for difficulties in the estimation [11]. When the breeds are in different SNP-QTL LD, the (co)variances of SNP effects are expected to differ along the genome, and across the breeds [11, 21, 22, 41].

### Genomic prediction considering breed origin of alleles

Models accounting for breed origin of each SNP allele, rather than genome-wide breed proportions estimated from pedigree or markers, have been proposed, and were shown to improve genomic predictions for simple 2 or 3-way crosses. Those studies applied either univariate whole genome regression models at the SNP level ignoring that the SNP effects might be correlated between the pure breeds [27, 55], or rather computationally demanding multi-trait genomic BLUP models with “partial” relationship matrices at the individual level [22, 56, 57]. It was claimed that considering genomic correlations between pure breeds had limited relevance in models for predicting crossbred performance [57].

Our results did not show any clear evidence of the benefit of accounting for correlations among the breeds when MIX data were used with the BOA approach, even for the breed (JER) with a small reference population, for which one would expect more gain in accuracy compared with breeds with a large reference population (HOL and RED). A possible explanation of the unobserved benefit for JER could be due to this breed being genetically distinct from HOL and RED [58], and therefore, the pattern of SNP effects along the genome being different from HOL and RED. In addition, the information in the data may be too weak to estimate correlations among the breeds. The MIX data also increased the within-breed data size to some extent, which may lower the importance of correlated information from other breeds [41]. For the scenario with a QTL effect correlation of 1.00, analysis with HOL+RED+JER+MIX was competitive with or even superior to the analysis using BOA without accounting for correlations between the breeds, particularly in predicting breeding values for individuals of MIX. This may be due to the MIX individuals being included in the reference population, which simply increases the data size in a combined analysis, whereas BOA with uncorrelated analysis uses only the information in a single breed (component).

**Fig. 4:**
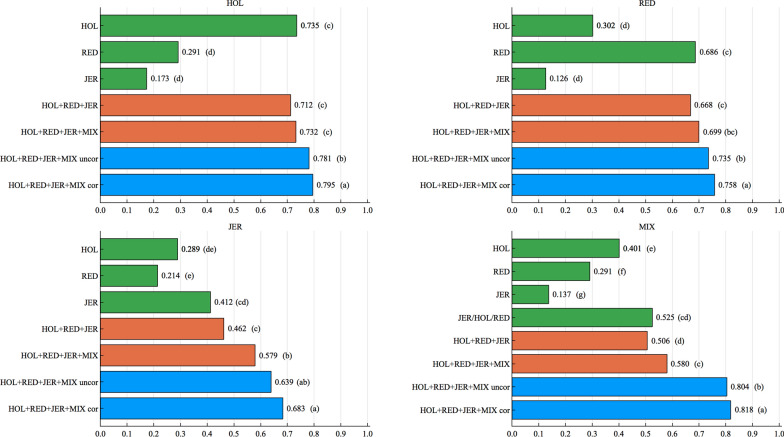
Accuracies (horizontal axis) for the low heritability trait ($h^2=0.05$) in the scenario with a QTL correlation of 0.50, using different data sets or models (vertical axis), when only the QTLs are considered with region size of 1 SNP. The predicted population is given on top of each plot. Letters in parentheses stand for the significance tests

The differences in LD pattern and phase persistency across different breeds [43] may result in marker effects being highly correlated in regions, where LD and SNP-QTL phase are constant between the breeds [41]. Hence, we have anticipated that correlations between the populations at the region level might improve the accuracy of genomic predictions, although the correlations at the whole genome level do not. In this study, the differences in accuracies from 100 SNPs and 1 SNP region sizes were generally negligible, whereas WG generally yielded the lowest accuracies. However, it is worth noting that the fixed-length of 100 SNPs as region size was arbitrarily chosen to give an insight on the impact of grouping SNPs in within-, across- and multi-breed genomic prediction accuracy, and there may exist other region sizes that yield higher accuracies than 100 SNPs. In analyses that aim at using correlations between breeds, such as the analysis using the BOA approach, the knowledge of the LD patterns and persistence of phase among the breeds may be useful for grouping SNPs.

van den Berg et al. [14] showed that prediction of breeding values and genomic correlations across populations can be more accurate if a carefully selected set of causal variants or SNPs that are very close to causal variants from sequencing data are used together with commercial SNP panels. Doing so may alleviate the issue of SNP-QTL LD being different in different breeds. In a recent study, Liu et al. [6] showed that integrating additional selected sequence variants to the standard 54K SNP chip led to significant improvements of reliabilities for the genomic evaluation of milk production traits in Danish Jersey. They reported that the benefits of using selected sequence variants in genomic prediction for milk and protein remained significant even in the scenario in which the largest reference population consisted of animals from Danish and US Jersey populations. In order to eliminate the impact of LD differences between the breeds on the comparison of accuracy when using the two BOA approaches (correlated and uncorrelated SNP effects), we ran additional analyses for the scenario with a QTL correlation of 0.50, and the low heritability trait $(h^2=0.05)$, using only the 250 QTL as SNPs and the region size of 1 SNP (see Additional file 2: Table S5). Accuracies using the BOA approach with correlated SNP effects between the breeds were higher than those with uncorrelated SNP effects between the breeds (Fig. 4). In light of these results and the results of [6, 14], one can argue that integrating selected sequence variants may be an efficient way of using correlated information from the breeds, and that in this case taking the correlation of SNP effects between breeds into account may allow for greater accuracy, in genomic evaluations with data from multiple purebred and admixed individuals using the BOA approach.

Estimation of the breed composition of individuals with admixed genomic background is of relevance for genomic prediction, because if it is not accounted for, it may lead to spurious estimates of SNP effects [59]. In real life applications, pedigree records and/or parentage validation can be used to distinguish purebred and admixed animals, but any error in the pedigree may lead to inaccurate consideration of individuals as pure or admixed [18]. Nevertheless, genomic prediction should rely on local ancestry (i.e., breed of origin) for each of the SNP alleles, rather than a genome-wide (global) ancestry computed from pedigree or markers [60]. Methods exist to estimate local ancestry in a population of admixed individuals (e.g., [61]). In this simulation study, breed origin of admixed individuals were known without error, but those could also be estimated from the data of purebred individuals. Due to mimicking a systematic crossing scheme with well-defined purebred individuals in our simulations, such estimates are expected to be highly accurate (Ana C. Guillenea, personal communication). However, for populations in which admixture is more complex, first one needs to find the number of pure breeds in the gene pool, and then to assign breed origin to each SNP allele for all animals in the population. This may introduce another source of error, and the models requiring breed origin of alleles, with or without accounting for correlations, may suffer from such errors to the extent where simply combining all available data (multiple pure and admixed breeds data) might become highly competitive. It was shown that a larger number of animals would be required to distinguish closely related breeds than to distinguish distantly related breeds [62], when the breed origin of an animal is needed to be inferred from the genotypic data. To the best of our knowledge, there is no information on the number of purebred animals required to correctly assign breed-origin of alleles of the crossbred animals.

Genetic background, as well as environment, alters the effects of QTL [10, 19]. Thus, the substitution effect of a QTL may not be stable between the admixed population and the pure breed it originates from. Although considering interactions between QTL and background in the simulation of substitution effects may be more realistic, it is not straightforward to make a decision on a realistic genetic background. Hence, in this study, such interactions were ignored, thereby breeding values of MIX were simulated using the substitution effects of the pure breeds. The BOA models used here can account for breed differences in allele substitution effects, however, they make no distinction on the basis of the genetic background, that is whether a QTL is expressed in a purebred or admixed individual’s genome. Further study may be needed to explore if and how interactions between QTL and background can be accommodated in BOA models, combining data from multiple pure breeds and admixed individuals.

### Genome scaling

Approximations for genomic prediction accuracy [3, 63] use the size of the reference population ($\textit{n}_{\text{R}}$), trait heritability (*h*^2^), and the effective number of chromosomal segments segregating in the population ($M_{e}$), where $M_{e}$ is a function of the genome length and the effective population size ($\textit{N}_{\text{e}}$). Following those studies [3, 63], within-breed prediction accuracy can be estimated with $\sqrt{\frac{h^2n_R}{(h^2n_R+M_e)}}$. In this study, only the first five chromosomes were simulated, which is roughly a quarter of the cattle genome. Those approximations suggest that, if we scale up the genome size (and the number of QTL) to that of the whole genome, and the size of the reference populations accordingly, our results will still hold, in within-breed predictions. For across-breed prediction, Wientjes et al. [64], suggested the use of $r_g\sqrt{\frac{h^2n_R}{(h^2n_R+M_e)}}$, where $r_g$ is the genetic correlation between breeds. They further suggested that $M_e$ values of 20,000 and 40,000 may be used when the populations are closely and distantly related, respectively. On the one hand, combining different breeds together will increase $N_e$ [65], and thereby $M_e$, requiring a larger reference population size to compensate this increase in $M_e$, to avoid a reduction in accuracy [36, 52]. On the other hand, models accounting for BOA make use of single-breed data, while taking advantage of an increase in $n_R$ by using data from admixed individuals. The BOA model that includes correlations further uses correlated information from other breeds. It is worth noting that those approximations assume a single homogenous target (validation) population.

## Conclusions

The aim of this simulation study was to provide a model allowing the inclusion of data from individuals with an admixed genetic background in genomic evaluations, while accounting for the differences in marker effects for each purebred population in the gene pool. Combining pure breeds’ and admixed population’s data in a multi-breed reference population was beneficial for the estimation of breeding values for pure breeds with a small reference population. For the admixed population, combining all available data (from purebred and admixed individuals) and realizing a combined genomic evaluation led to higher accuracies than considering BOA for selection candidates only and using breed-specific SNP effects estimated separately in each pure breed. Including data from admixed individuals in the reference population of multiple breeds by considering BOA, accuracies were further improved. Our findings are relevant for breeding programs in which crossbreeding is systematically applied (e.g., ProCROSS system, http://www.procross.info), and also for populations involving different subpopulations between which exchange of genetic material has become routine practice (e.g., Nordic Red dairy cattle).

## Supplementary information

**Additional file 1: Figure S1.** Plot of the first two principle components from the PCA analysis of the genomic relationship matrix.

**Additional file 2: Table S1.** Accuracies for purebred individuals for a trait with high heritability h^2^ = 0.40. **Table S2.** Accuracies for purebred individuals for a trait with low heritability h^2^ = 0.05. **Table S3.** Accuracies for admixed individuals (MIX) for a trait with high heritability h^2^ = 0.40. **Table S4.** Accuracies for admixed individuals (MIX) for a trait with low heritability h^2^ = 0.05. **Table S5.** Accuracy for the low heritability h^2^ = 0.05 trait, using only 250 QTL and region size of 1 SNP.

## Data Availability

The datasets used during the current study are available from the corresponding author on reasonable request.
