# Supplementary material for: Genomic prediction using a reference population of multiple pure breeds and admixed individuals
Source: Genet Sel Evol. 2021 May 31;53:46. doi: 10.1186/s12711-021-00637-y (PMC8168010; doi:10.1186/s12711-021-00637-y)
Supplement: Supplementary file 1 — Additional file 1: Figure S1. Plot of the first two principle components from the PCA analysis of the genomic relationship matrix. [file 12711_2021_637_MOESM1_ESM.pdf]

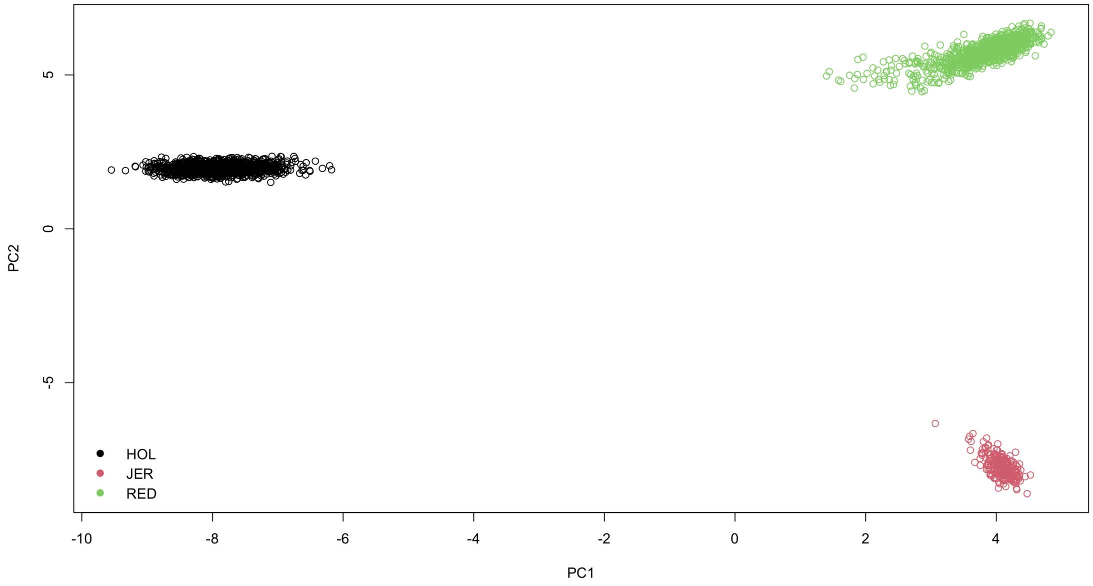

Figure S1. Plot of the first two principle components from the PCA analysis of genomic relationship matrix. Genomic relationships were computed as described in [1], and analysis were carried out using R function prcomp() [2].

References

[1] Wientjes Y, Veerkamp R, Bijma P, Bovenhuis H, Schrooten C, Calus MPL. Empirical and deterministic accuracies of across-population genomic prediction. Genet Sel Evol. 2015;47:5.

[2] R Core Team: R: A Language and Environment for Statistical Computing. R Foundation for Statistical Computing, Vienna, Austria, 2020.
