# Supplementary material for: Genomic prediction using a reference population of multiple pure breeds and admixed individuals
Source: Genet Sel Evol. 2021 May 31;53:46. doi: 10.1186/s12711-021-00637-y (PMC8168010; doi:10.1186/s12711-021-00637-y)
Supplement: Supplementary file 2 — Additional file 2: Table S1. Accuracies for purebred individuals for a trait with high heritability h2 = 0.40. Table S2. Accuracies for purebred individuals for a trait with low heritability h2 = 0.05. Table S3. Accuracies for admixed individuals (MIX) for a trait with high heritability h2 = 0.40. Table S4. Accuracies for admixed individuals (MIX) for a trait with low heritability h2 = 0.05. Table S5. Accuracy for the low heritability h2 = 0.05 trait, using only 250 QTL and region size of 1 SNP. [file 12711_2021_637_MOESM2_ESM.pdf]

Table S1: Accuracies for purebred individuals for a trait with high heritability $\left(h^2 = 0.40\right)$

			**HOL**			**RED**			**JER**		
**Correlation[1]**	**Training[2]**	**Data/Region Size[3]**	**1 SNP**	**100 SNPs**	**WG**	**1 SNP**	**100 SNPs**	**WG**	**1 SNP**	**100 SNPs**	**WG**
1.00	Pure	HOL	$_{b}0.771^{b}$	$_{b}0.780^{a}$	$_{b}0.760^{c}$	$_{d}0.128^{a}$	$_{d}0.095^{a}$	$_{d}0.080^{b}$	$_{d}0.120^{a}$	$_{c}0.059^{b}$	$_{c}0.036^{c}$
		RED	$_{c}0.170^{a}$	$_{c}0.162^{a}$	$_{c}0.127^{b}$	$_{c}0.751^{b}$	$_{c}0.757^{a}$	$_{bc}0.741^{c}$	$_{d}0.170^{a}$	$_{c}0.123^{b}$	$_{c}0.111^{b}$
		JER	$_{c}0.042^{a}$	$_{d}0.035^{a}$	$_{d}0.040^{a}$	$_{d}0.060^{a}$	$_{d}0.057^{a}$	$_{d}0.057^{a}$	$_{c}0.643^{b}$	$_{b}0.652^{a}$	$_{b}0.638^{c}$
	Combined	HOL+RED+JER	$_{b}0.777^{a}$	$_{b}0.779^{a}$	$_{b}0.757^{b}$	$_{c}0.749^{a}$	$_{c}0.752^{a}$	$_{c}0.730^{b}$	$_{bc}0.652^{a}$	$_{b}0.646^{a}$	$_{b}0.617^{b}$
		HOL+RED+JER+MIX	$_{a}0.802^{a}$	$_{a}0.803^{a}$	$_{a}0.781^{b}$	$_{b}0.774^{a}$	$_{b}0.777^{a}$	$_{ab}0.754^{b}$	$_{a}0.732^{a}$	$_{a}0.734^{a}$	$_{a}0.701^{b}$
	BOA	HOL+RED+JER+MIX uncor	$_{a}0.796^{b}$	$_{a}0.804^{a}$	$_{a}0.782^{c}$	$_{ab}0.776^{b}$	$_{ab}0.782^{a}$	$_{a}0.763^{c}$	$_{ab}0.723^{b}$	$_{a}0.738^{a}$	$_{a}0.713^{c}$
		HOL+RED+JER+MIX cor	$_{a}0.803^{a}$	$_{a}0.805^{a}$	$_{a}0.783^{b}$	$_{a}0.776^{a}$	$_{a}0.779^{a}$	$_{a}0.758^{b}$	$_{a}0.734^{a}$	$_{a}0.740^{a}$	$_{a}0.703^{b}$
0.50	Pure	HOL	$_{b}0.785^{b}$	$_{b}0.790^{a}$	$_{b}0.774^{c}$	$_{d}0.101^{a}$	$_{d}0.085^{ab}$	$_{d}0.073^{b}$	$_{d}0.102^{a}$	$_{d}0.073^{b}$	$_{e}0.067^{b}$
		RED	$_{d}0.131^{ab}$	$_{d}0.129^{a}$	$_{d}0.116^{b}$	$_{b}0.747^{b}$	$_{b}0.754^{a}$	$_{b}0.736^{c}$	$_{d}0.079^{a}$	$_{d}0.070^{a}$	$_{e}0.043^{b}$
		JER	$_{d}0.030^{a}$	$_{d}0.039^{a}$	$_{d}0.029^{a}$	$_{d}0.023^{a}$	$_{d}0.025^{a}$	$_{d}0.024^{a}$	$_{c}0.629^{ab}$	$_{c}0.635^{a}$	$_{cd}0.624^{b}$
	Combined	HOL+RED+JER	$_{c}0.773^{a}$	$_{c}0.774^{a}$	$_{c}0.761^{b}$	$_{c}0.729^{a}$	$_{c}0.732^{a}$	$_{c}0.716^{b}$	$_{c}0.599^{a}$	$_{c}0.587^{b}$	$_{d}0.590^{ab}$
		HOL+RED+JER+MIX	$_{bc}0.782^{a}$	$_{bc}0.782^{a}$	$_{bc}0.771^{b}$	$_{bc}0.742^{a}$	$_{bc}0.747^{a}$	$_{bc}0.732^{b}$	$_{b}0.695^{a}$	$_{b}0.690^{a}$	$_{bc}0.675^{b}$
	BOA	HOL+RED+JER+MIX uncor	$_{a}0.804^{a}$	$_{a}0.807^{a}$	$_{a}0.792^{b}$	$_{a}0.774^{b}$	$_{a}0.780^{a}$	$_{a}0.762^{c}$	$_{ab}0.730^{a}$	$_{a}0.738^{a}$	$_{ab}0.712^{b}$
		HOL+RED+JER+MIX cor	$_{a}0.807^{a}$	$_{a}0.808^{a}$	$_{a}0.793^{b}$	$_{a}0.775^{a}$	$_{a}0.778^{a}$	$_{a}0.761^{b}$	$_{a}0.737^{a}$	$_{a}0.737^{a}$	$_{a}0.713^{b}$
0.25	Pure	HOL	$_{b}0.782^{b}$	$_{b}0.788^{a}$	$_{b}0.772^{c}$	$_{d}0.053^{a}$	d0.041^{ab}	$_{d}0.034^{b}$	$_{d}0.089^{a}$	$_{d}0.066^{ab}$	$_{d}0.058^{b}$
		RED	$_{d}0.080^{a}$	$_{d}0.081^{a}$	$_{d}0.080^{a}$	$_{b}0.742^{b}$	$_{b}0.750^{a}$	$_{b}0.730^{c}$	$_{d}0.017^{a}$	$_{d}0.012^{a}$	$_{d}0.003^{a}$
		JER	$_{d}0.026^{a}$	$_{d}0.034^{a}$	$_{d}0.025^{a}$	$_{d}0.017^{a}$	$_{d}0.014^{a}$	$_{d}0.019^{a}$	$_{bc}0.632^{a}$	$_{b}0.639^{a}$	$_{bc}0.627^{b}$
	Combined	HOL+RED+JER	$_{c}0.764^{a}$	$_{c}0.766^{a}$	$_{c}0.755^{b}$	$_{c}0.716^{b}$	$_{c}0.721^{a}$	$_{c}0.705^{c}$	$_{c}0.587^{a}$	$_{c}0.573^{b}$	$_{c}0.581^{ab}$
		HOL+RED+JER+MIX	$_{bc}0.769^{a}$	$_{c}0.768^{ab}$	$_{bc}0.761^{b}$	$_{bc}0.725^{a}$	$_{bc}0.731^{a}$	$_{bc}0.718^{b}$	$_{b}0.689^{a}$	$_{b}0.680^{b}$	$_{b}0.667^{c}$
	BOA	HOL+RED+JER+MIX uncor	$_{a}0.803^{a}$	$_{a}0.805^{a}$	$_{a}0.790^{b}$	$_{a}0.771^{b}$	$_{a}0.777^{a}$	$_{a}0.759^{c}$	$_{a}0.735^{a}$	$_{a}0.743^{a}$	$_{a}0.716^{b}$
		HOL+RED+JER+MIX cor	$_{a}0.805^{a}$	$_{a}0.806^{a}$	$_{a}0.790^{b}$	$_{a}0.772^{a}$	$_{a}0.775^{a}$	$_{a}0.758^{b}$	$_{a}0.740^{a}$	$_{a}0.741^{a}$	$_{a}0.715^{b}$

[1] Correlation of simulated QTL effects. Different alphabets mean significantly different values at a Type 1 error rate of 0.05 with Bonferroni correction. Subscripts (within region size) and superscripts (within data) stand for comparisons within column and row, respectively, for each correlation scenario.

[2] The methods classified based on the data and model used to estimate SNP effects

[3] Data: Data included in reference population. Region Size: Number of SNPs assigned the same variance.

Table S2: Accuracies for purebred individuals for a trait with low heritability $(h^2 = 0.05)$

			HOL			RED			JER		
**Correlation**[1]	**Training**[2]	**Data/Region Size**[3]	**1 SNP**	**100 SNPs**	**WG**	**1 SNP**	**100 SNPs**	**WG**	**1 SNP**	**100 SNPs**	**WG**
1.00	Pure	HOL	$_{c}0.469^{a}$	$_{c}0.470^{ab}$	$_{a}0.466^{b}$	$_{e}0.019^{a}$	$_{d}0.010^{b}$	$_{d}0.022^{ab}$	$_{c}\text{-}0.023^{a}$	$_{c}\text{-}0.023^{a}$	$_{b}\text{-}0.021^{a}$
		RED	$_{d}0.055^{a}$	$_{d}0.047^{a}$	$_{b}0.047^{a}$	$_{bd}0.421^{a}$	$_{bc}0.420^{ab}$	$_{bc}0.417^{b}$	$_{c}0.044^{a}$	$_{c}0.043^{a}$	$_{b}0.045^{a}$
		JER	$_{d}\text{-}0.008^{a}$	$_{d}\text{-}0.002^{a}$	$_{b}0.002^{a}$	$_{e}0.036^{a}$	$_{d}0.035^{a}$	$_{d}0.040^{a}$	$_{ab}0.319^{a}$	$_{ab}0.319^{a}$	$_{a}0.321^{a}$
	Combined	HOL+RED+JER	$_{bc}0.467^{a}$	$_{c}0.472^{a}$	$_{a}0.463^{a}$	$_{cd}0.415^{a}$	$_{c}0.411^{a}$	$_{c}0.408^{a}$	$_{b}0.291^{a}$	$_{b}0.294^{a}$	$_{a}0.289^{a}$
		HOL+RED+JER+MIX	$_{ab}0.501^{a}$	$_{b}0.503^{ab}$	$_{a}0.494^{b}$	$_{ab}0.453^{a}$	$_{ab}0.450^{ab}$	$_{ab}0.445^{b}$	$_{ab}0.389^{a}$	$_{a}0.385^{a}$	$_{a}0.381^{a}$
	BOA	HOL+RED+JER+MIX uncor	$_{abc}0.500^{a}$	$_{abc}0.501^{a}$	$_{a}0.497^{a}$	$_{ac}0.451^{a}$	$_{a}0.451^{ab}$	$_{a}0.447^{b}$	$_{ab}0.387^{a}$	$_{ab}0.382^{a}$	$_{a}0.383^{a}$
		HOL+RED+JER+MIX cor	$_{a}0.503^{a}$	$_{a}0.506^{ab}$	$_{a}0.495^{b}$	$_{ab}0.454^{a}$	$_{ab}0.450^{ab}$	$_{ab}0.444^{b}$	$_{a}0.392^{a}$	$_{a}0.389^{a}$	$_{a}0.382^{a}$
0.50	Pure	HOL	$_{b}0.476^{a}$	$_{a}0.478^{a}$	$_{a}0.474^{a}$	$_{d}0.028^{a}$	$_{c}0.024^{a}$	$_{c}0.030^{a}$	$_{c}0.007^{a}$	$_{c}0.017^{a}$	$_{c}0.010^{a}$
		RED	$_{c}0.068^{a}$	$_{b}0.080^{a}$	$_{b}0.071^{a}$	$_{c}0.410^{a}$	$_{b}0.412^{a}$	$_{b}0.410^{a}$	$_{c}0.024^{a}$	$_{c}0.030^{a}$	$_{c}0.028^{a}$
		JER	$_{c}0.018^{a}$	$_{b}0.015^{a}$	$_{b}0.012^{a}$	$_{d}0.016^{ab}$	$_{c}0.013^{b}$	$_{c}0.026^{a}$	$_{b}0.260^{a}$	$_{b}0.260^{a}$	$_{b}0.264^{a}$
	Combined	HOL+RED+JER	$_{b}0.466^{a}$	$_{a}0.467^{a}$	$_{a}0.464^{a}$	$_{c}0.399^{a}$	$_{b}0.393^{a}$	$_{b}0.397^{a}$	$_{b}0.240^{a}$	$_{b}0.238^{a}$	$_{b}0.243^{a}$
		HOL+RED+JER+MIX	$_{b}0.487^{a}$	$_{a}0.483^{a}$	$_{a}0.482^{a}$	$_{bc}0.433^{a}$	$_{b}0.432^{a}$	$_{ab}0.432^{a}$	$_{a}0.378^{a}$	$_{a}0.374^{a}$	$_{a}0.372^{a}$
	BOA	HOL+RED+JER+MIX uncor	$_{ab}0.502^{a}$	$_{a}0.498^{a}$	$_{a}0.499^{a}$	$_{ab}0.457^{a}$	$_{a}0.459^{a}$	$_{a}0.455^{a}$	$_{a}0.383^{a}$	$_{a}0.388^{a}$	$_{a}0.384^{a}$
		HOL+RED+JER+MIX cor	$_{a}0.504^{a}$	$_{a}0.502^{a}$	$_{a}0.499^{a}$	$_{a}0.455^{a}$	$_{a}0.457^{a}$	$_{a}0.450^{a}$	$_{a}0.390^{a}$	$_{a}0.394^{a}$	$_{a}0.382^{a}$
0.25	Pure	HOL	$_{ab}0.473^{a}$	$_{a}0.474^{a}$	$_{ab}0.471^{a}$	$_{c}0.012^{a}$	$_{d}0.007^{a}$	$_{e}0.014^{a}$	$_{c}0.005^{a}$	$_{c}0.016^{a}$	$_{c}0.007^{a}$
		RED	$_{c}0.050^{a}$	$_{b}0.059^{a}$	$_{c}0.054^{a}$	$_{b}0.407^{a}$	$_{b}0.409^{a}$	$_{c}0.407^{a}$	$_{c}\text{-}0.006^{a}$	$_{c}\text{-}0.006^{a}$	$_{c}0.000^{a}$
		JER	$_{c}0.017^{a}$	$_{b}0.014^{a}$	$_{c}0.012^{a}$	$_{c}0.024^{b}$	$_{d}0.017\text{b}$	$_{e}0.032^{a}$	$_{b}0.260^{a}$	$_{b}0.260^{a}$	$_{b}0.265^{a}$
	Combined	HOL+RED+JER	$_{b}0.459^{a}$	$_{a}0.458^{a}$	$_{b}0.458^{a}$	$_{b}0.392^{a}$	$_{c}0.385^{a}$	$_{d}0.391^{a}$	$_{b}0.228^{a}$	$_{b}0.226^{a}$	$_{b}0.233^{a}$
		HOL+RED+JER+MIX	$_{b}0.476^{a}$	$_{a}0.472^{a}$	$_{b}0.473^{a}$	$_{b}0.423^{a}$	$_{bc}0.417^{a}$	$_{bcd}0.424^{a}$	$_{a}0.372^{a}$	$_{a}0.364^{a}$	$_{a}0.368^{a}$
	BOA	HOL+RED+JER+MIX uncor	$_{a}0.499^{a}$	$_{a}0.494^{a}$	$_{a}0.495^{a}$	$_{a}0.454^{b}$	$_{a}0.458^{a}$	$_{ab}0.453^{ab}$	$_{a}0.388^{a}$	$_{a}0.392^{a}$	$_{a}0.389^{a}$
		HOL+RED+JER+MIX cor	$_{a}0.501^{a}$	$_{a}0.497^{a}$	$_{ab}0.495^{a}$	$_{a}0.454^{ab}$	$_{a}0.458^{a}$	$_{a}0.451^{b}$	$_{a}0.393^{a}$	$_{a}0.397^{a}$	$_{a}0.384^{a}$

[1] Correlation of simulated QTL effects. Different alphabets mean significantly different values at a Type 1 error rate of 0.05 with Bonferroni correction. Subscripts (within region size) and superscripts (within data) stand for comparisons within column and row, respectively, for each correlation scenario.

[2] The methods classified based on the data and model used to estimate SNP effects

[3] Data: Data included in reference population. Region Size: Number of SNPs assigned the same variance.

Table S3: Accuracies for admixed individuals (MIX) for a trait with high heritability $\left(h^2 = 0.40\right)$

			MIX		
**Correlation[1]**	**Training[2]**	**Data/Region Size[3]**	**1 SNP**	**100 SNPs**	**WG**
1.00	Pure	HOL	$_{e}0.578^{a}$	$_{e}0.576^{a}$	$_{e}0.562^{b}$
		RED	$_{f}0.395^{a}$	$_{f}0.395^{a}$	$_{f}0.372^{b}$
		JER	$_{g}0.178^{a}$	$_{g}0.179^{a}$	$_{g}0.175^{a}$
		HOL/RED/JER	$_{d}0.687^{b}$	$_{d}0.704^{a}$	$_{d}0.685^{b}$
	Combined	HOL+RED+JER	$_{c}0.732^{a}$	$_{c}0.732^{a}$	$_{c}0.710^{b}$
		HOL+RED+JER+MIX	$_{a}0.794^{a}$	$_{a}0.798^{a}$	$_{a}0.775^{b}$
	BOA	HOL+RED+JER+MIX uncor	$_{b}0.772^{b}$	$_{b}0.782^{a}$	$_{b}0.762^{c}$
		HOL+RED+JER+MIX cor	$_{a}0.794^{b}$	$_{a}0.799^{a}$	$_{a}0.776^{c}$
0.50	Pure	HOL	$_{d}0.411^{a}$	$_{d}0.415^{a}$	$_{d}0.410^{a}$
		RED	$_{e}0.275^{a}$	$_{e}0.274^{a}$	$_{e}0.268^{a}$
		JER	$_{f}0.114^{a}$	$_{f}0.117^{a}$	$_{f}0.114^{a}$
		HOL/RED/JER	$_{c}0.531^{a}$	$_{c}0.533^{ab}$	$_{c}0.521^{b}$
	Combined	HOL+RED+JER	$_{c}0.560^{a}$	$_{c}0.556^{ab}$	$_{c}0.554^{b}$
		HOL+RED+JER+MIX	$_{b}0.827^{a}$	$_{b}0.824^{a}$	$_{b}0.817^{b}$
	BOA	HOL+RED+JER+MIX uncor	$_{a}0.876^{a}$	$_{a}0.877^{a}$	$_{a}0.870^{b}$
		HOL+RED+JER+MIX cor	$_{a}0.877^{a}$	$_{a}0.877^{a}$	$_{a}0.870^{b}$
0.25	Pure	HOL	$_{d}0.358^{a}$	$_{d}0.363^{a}$	$_{d}0.360^{a}$
		RED	$_{e}0.231^{a}$	$_{e}0.231^{a}$	$_{e}0.231^{a}$
		JER	$_{e}0.106^{a}$	$_{e}0.109^{a}$	$_{e}0.106^{a}$
		HOL/RED/JER	$_{c}0.482^{a}$	$_{c}0.482^{ab}$	$_{c}0.469^{b}$
	Combined	HOL+RED+JER	$_{c}0.513^{a}$	$_{c}0.509^{ab}$	$_{c}0.509^{b}$
		HOL+RED+JER+MIX	$_{b}0.840^{a}$	$_{b}0.837^{a}$	$_{b}0.829^{b}$
	BOA	HOL+RED+JER+MIX uncor	$_{a}0.897^{a}$	$_{a}0.898^{a}$	$_{a}0.892^{b}$
		HOL+RED+JER+MIX cor	$_{a}0.897^{a}$	$_{a}0.897^{a}$	$_{a}0.892^{b}$

[1] Correlation of simulated QTL effects. Different alphabets mean significantly different values at a Type 1 error rate of 0.05 with Bonferroni correction. Subscripts (within region size) and superscripts (within data) stand for comparisons within column and row, respectively, for each correlation scenario.

[2] The methods classified based on the data and model used to estimate SNP effects

[3] Data: Data included in reference population. Region Size: Number of SNPs assigned the same variance.

Table S4: Accuracies for admixed individuals (MIX) for a trait with low heritability $\left(h^2 = 0.05\right)$

			MIX		
**Correlation[1]**	**Training[2]**	**Data/Region Size[3]**	**1 SNP**	**100 SNPs**	**WG**
1.00	Pure	HOL	${}_{d}0.363^{a}$	${}_{c}0.364^{a}$	${}_{c}0.362^{a}$
		RED	${}_{e}0.229^{a}$	${}_{d}0.225^{ab}$	${}_{d}0.221^{b}$
		JER	${}_{f}0.075^{a}$	${}_{e}0.077^{a}$	${}_{e}0.078^{a}$
		HOL/RED/JER	${}_{c}0.433^{a}$	${}_{b}0.436^{a}$	${}_{b}0.429^{a}$
	Combined	HOL+RED+JER	${}_{b}0.449^{a}$	${}_{b}0.449^{a}$	${}_{b}0.443^{a}$
		HOL+RED+JER+MIX	${}_{a}0.503^{a}$	${}_{a}0.501^{ab}$	${}_{a}0.496^{b}$
	BOA	HOL+RED+JER+MIX uncor	${}_{bcd}0.430^{ab}$	${}_{bc}0.434^{a}$	${}_{bc}0.424^{b}$
		HOL+RED+JER+MIX cor	${}_{a}0.500^{a}$	${}_{a}0.499^{a}$	${}_{a}0.493^{a}$
0.50	Pure	HOL	${}_{d}0.256^{a}$	${}_{d}0.256^{a}$	${}_{d}0.255^{a}$
		RED	${}_{e}0.157^{a}$	${}_{e}0.162^{a}$	${}_{e}0.155^{a}$
		JER	${}_{f}0.043^{a}$	${}_{f}0.043^{a}$	${}_{e}0.043^{a}$
		HOL/RED/JER	${}_{cd}0.303^{ab}$	${}_{cd}0.308^{a}$	${}_{cd}0.299^{b}$
	Combined	HOL+RED+JER	${}_{c}0.356^{a}$	${}_{c}0.356^{a}$	${}_{c}0.353^{a}$
		HOL+RED+JER+MIX	${}_{b}0.557^{a}$	${}_{b}0.546^{a}$	${}_{b}0.550^{a}$
	BOA	HOL+RED+JER+MIX uncor	${}_{a}0.674^{ab}$	${}_{a}0.676^{a}$	${}_{a}0.672^{b}$
		HOL+RED+JER+MIX cor	${}_{a}0.672^{a}$	${}_{a}0.673^{a}$	${}_{a}0.669^{a}$
0.25	Pure	HOL	${}_{de}0.226^{a}$	${}_{de}0.227^{a}$	${}_{de}0.226^{a}$
		RED	${}_{ef}0.136^{a}$	${}_{ef}0.138^{a}$	${}_{ef}0.134^{a}$
		JER	${}_{f}0.040^{a}$	${}_{f}0.041^{a}$	${}_{f}0.041^{a}$
		HOL/RED/JER	${}_{cd}0.271^{ab}$	${}_{cd}0.277^{a}$	${}_{cd}0.266^{b}$
	Combined	HOL+RED+JER	${}_{c}0.339^{a}$	${}_{c}0.338^{a}$	${}_{c}0.336^{a}$
		HOL+RED+JER+MIX	${}_{b}0.587^{a}$	${}_{b}0.573^{b}$	${}_{b}0.575^{ab}$
	BOA	HOL+RED+JER+MIX uncor	${}_{a}0.729^{a}$	${}_{a}0.730^{ab}$	${}_{a}0.727^{b}$
		HOL+RED+JER+MIX cor	${}_{a}0.728^{a}$	${}_{a}0.728^{a}$	${}_{a}0.725^{a}$

[1] Correlation of simulated QTL effects. Different alphabets mean significantly different values at a Type 1 error rate of 0.05 with Bonferroni correction. Subscripts (within region size) and superscripts (within data) stand for comparisons within column and row, respectively, for each correlation scenario.

[2] The methods classified based on the data and model used to estimate SNP effects

[3] Data: Data included in reference population. Region Size: Number of SNPs assigned the same variance.

Table S5: Accuracy for the low heritability $(h^2 = 0.05)$ trait, using only 250 QTL and region size of 1 SNP

Correlation[1]	Training[2]	Data[3]/Validation population	HOL	RED	JER	MIX
0.50	Pure	HOL	$_{c}0.735$	$_{d}0.302$	$_{de}0.289$	$_{e}0.401$
		RED	$_{d}0.291$	$_{c}0.686$	$_{e}0.214$	$_{f}0.291$
		JER	$_{d}0.173$	$_{d}0.126$	$_{cd}0.412$	$_{g}0.137$
		HOL/RED/JER				$_{cd}0.525$
	Combined	HOL+RED+JER	$_{c}0.712$	$_{c}0.668$	$_{c}0.462$	$_{d}0.506$
		HOL+RED+JER+MIX	$_{c}0.732$	$_{bc}0.699$	$_{b}0.579$	$_{c}0.580$
	BOA	HOL+RED+JER+MIX uncor	$_{b}0.781$	$_{b}0.735$	$_{ab}0.639$	$_{b}0.804$
		HOL+RED+JER+MIX cor	$_{a}0.795$	$_{a}0.758$	$_{a}0.683$	$_{a}0.818$

[1] Correlation of simulated QTL effects. Different alphabets mean significantly different values at a Type 1 error rate of 0.05 with Bonferroni correction. Subscripts (region size of 1 SNP) and superscripts (within data) stand for comparisons within column and row, respectively, for each correlation scenario.

[2] The methods classified based on the data and model used to estimate QTL effects

[3] Data: Data included in reference population.
